# Egg banks in hypersaline lakes of the South-East Europe

**DOI:** 10.1186/1746-1448-5-3

**Published:** 2009-03-17

**Authors:** Salvatore Moscatello, Genuario Belmonte

**Affiliations:** 1 Laboratory of Zoogeography and Fauna, Department of Biological and Environmental Sciences and Technologies, University of Salento, Via Prov.le Lecce-Monteroni, 73100 Lecce, Italy

## Abstract

The cyst banks of 6 coastal hypersaline lakes of South-East Europe have been investigated. The study concerned the bottom sediments of Khersonesskoe and Koyashskoe lakes in the Crimea (Ukraine), Nartë saltworks (Albania), Vecchia Salina at Torre Colimena (Apulia, Italy), Pantano Grande and Pantano Roveto at Vendicari (Sicily, Italy). A total of 19 cyst types were recognised. The cyst banks of lakes were found to be well separated in the representation derived from a statistical multivariate data analysis. For all the lakes examined a comparison was possible between the resting community in sediments (cyst bank) and the active one in the water. The cyst banks contained more species than those recorded over a multi-year sampling effort in the water column. The study of cyst hatching, performed on 5 cyst types under lab conditions, demonstrated that cysts do not hatch under the same conditions. Furthermore, each cyst type shows a wide range of preferential hatching conditions, which allow us to confirm the ecological generalism of salt lake species.

## Background

Hypersaline lakes have been stimulating the interest of biologists for a long time, principally on account of their simple biological composition [1]. Over the last decades, the scientific literature on salt lakes has grown with the appearance of a large number of papers, and new research directions, e.g. ecology of hypersaline environments [2], agriculture applied to saline lakes [3], and saline lake conservation and management [4]. Recently, research on saline lake species assemblages has developed in scope and intensity. A general rule on the composition of biological communities has been recognised with an inverse correlation existing between species richness and salinity [1], with a small number of trophic roles each represented by just one species [5]. The species composition differs in saline waters of different chemistry [6,7], being characterized by marine originated fauna with *Artemia *shrimps where Cl^-1 ^anions where dominant, and by non marine Calanoida copepods (family Diaptomidae) where SO_4_^-2 ^is the main anion.

Thanks to the ability of many species to produce resting stages, coupled with long lasting periods of lacking of suitable conditions for active populations, hypersaline lakes hide a potential biodiversity (in terms of dormant species) which cannot be investigated by collecting organisms only from the water column. In fact, commonly during both dry-hot and freezing-cold seasons, most species stay in a dormant stage in the bottom sediments. The rest could even last for years if suitable conditions do not return (e.g. in the case of progressively rising salinity), giving a progressive reduction of active biodiversity (as in the case of the Aral Sea, see [8]). In these cases only a small portion of the biodiversity is expressed, the majority of species being temporarily resting as cysts in the sediments. Even on the return of suitable conditions, only a portion of the cysts produced will hatch, while the other ones go to storage the persistent cyst bank, where they can remain viable for decades or longer [9]. A recent study [10] demonstrated that the number of cyst types in the sediment of a salt lake were more than those of organisms present in the water (and carefully studied with a year round sample effort).

In studies carried out on cyst banks, generally most viable (responsive) cysts occur in the upper centimetres, although variations occur along the sediment column [11]. The accumulation of cysts of different species, generations and genotypes with variable germination rates, results in a complex assemblage which supplies the active community, representing the resilience of each system and the core of the so-called Supply Vertical Ecology [12]. Some recent opinions stress the opportunity to adopt integrated investigation procedures (collecting both from the water column and from the sediments) to obtain information as complete as possible on the species composition of a periodically stressed environment [10,13].

Based on these points, sampling stations for long-term observation of biology and correlated variables (meteorology and hydrology) could be established in key hypersaline lakes to integrate data with geographic information with the aim to monitor changes in saline lake areas [4].

This paper aims to spread the practice of the cyst bank analysis in geographically extended investigations on the biodiversity of water bodies. In fact the great advantage that the cyst bank analysis offers reducing the sampling effort would be carefully considered in geographically extended studies. In addition, any studies on resting stages will add information for the understanding of population dynamics of hypersaline plankters, leading to a significant improvement of our knowledge of the basic functioning of hypersaline lake systems.

## Results

**Figure 1:**
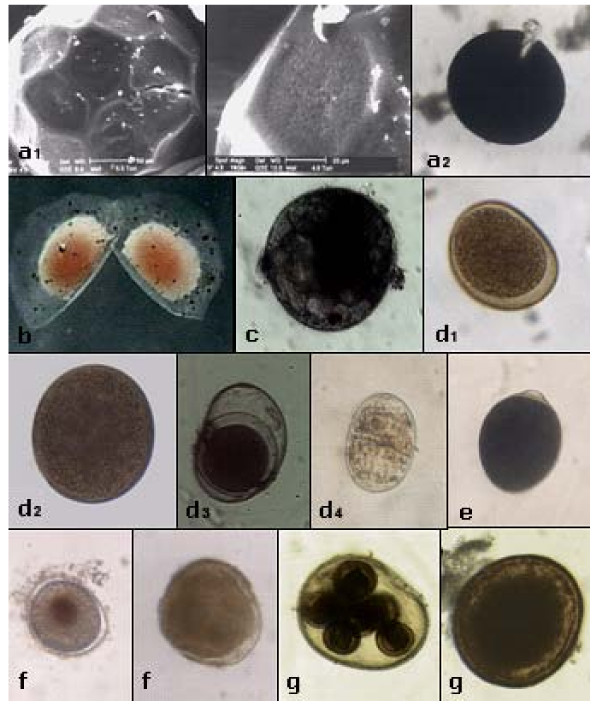
**Resting stages (cysts) of plankton organisms collected in the sediment of the studied hypersaline lakes**. In some groups the outer sculpturing may help in *taxon *identification: a) resting eggs of large branchiopods (a1 *Phallocryptus spinosa*; a2 *Artemia parthenogenetica*) (range 200–400 μm); b) ephippium of anomopod cladocerans (*Moina salina*) holding one resting egg (range 0,5–1 mm); c) types of spherical resting eggs in calanoid copepods (range 80–200 μm); d) types of monogonont rotifers resting eggs (d2 *Hexarthra fennica*; d3 *Brachionus *sp.; d4 *Ptygura *sp.)(range 80–200 μm); (e) flask-shaped cyst in ciliates (*Fabrea salina*) (range 80–200 μm); f) undetermined spherical resting eggs (range 80–200 μm); g) type of spherical resting eggs in turbellarians (range 80–200 μm).

A total of 19 cyst morphotypes were found (only 13 are given in Figure 1) in the 6 hypersaline lakes studied. Seven of them have been identified at the genus level, also due to the successful hatching experiments carried out in laboratory conditions. On the average, the number of cyst morphotypes extracted from the sediment cores was more than double the number of the active organisms found in the plankton over the study time [see Additional file 1]. The richest cyst bank was that of Vecchia Salina (17 morphotypes), and the poorest was that of lake Koyashskoe (5 morphotypes).

**Figure 2:**
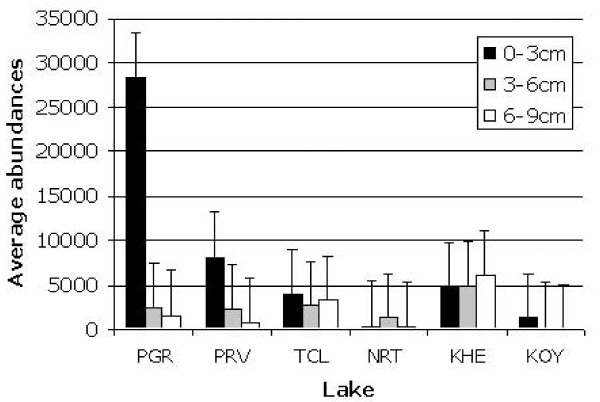
**Abundance of cysts in the sediment layers of the investigated lakes (number of cysts/100 cm^3^); PGR = *Pantano Grande *pool at Vendicari (Sicily, Italy); PRV = *Pantano Roveto *pool at Vendicari (Sicily, Italy); TCL = Torre Colimena (*Vecchia Salina*) pond (Apulia, Italy); NRT = *Nartë *saltworks (Vlorë, Albania); KHE = *Khersonesskoe *lake (Crimean Peninsula, Ukraine); KOY = *Koyashskoe *lake (Crimean Peninsula, Ukraine)**.

The most common cyst types were Turbellaria cocoons, which were present in all the sediment samples. Regarding abundances, the cyst bank of lake Pantano Grande at Vendicari (Sicily, Italy) was the richest, and Koyashskoe was the poorest (average on three multilayered cores from each lake, 10812 and 504 cysts/100 cm^3 ^respectively). Generally the cyst content in upper sediment layers was higher than that in the lower ones, but in the Vecchia Salina (Apulia, Italy) the difference among the three layers considered (0–3, 3–6, and 6–9 cm) was not evident (3924, 2634, and 3284 cysts/100 cm^3 ^respectively) and in Khersonesskoe the trend was reversed (4747, 4937, and 6088 cysts/100 cm^3^) (Figure 2).

**Figure 3:**
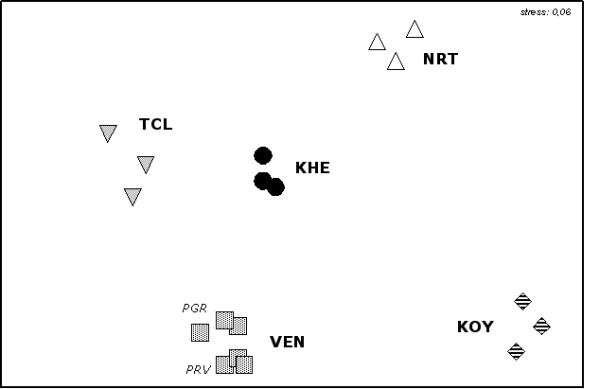
**MDS plot showing differences of cyst composition among "seed banks" from the studied lakes**. The absolute densities of each resting morphotypes were fourth root transformed. VEN = Vendicari pools (Sicily, Italy); TCL = Torre Colimena (*Vecchia Salina*) pond (Apulia, Italy); NRT = *Nartë *saltworks (Vlorë, Albania); KHE = *Khersonesskoe *lake (Crimean Peninsula, Ukraine); KOY = *Koyashskoe *lake (Crimean Peninsula, Ukraine).

Some species as *Moina salina *(Cladocera), *Artemia urmiana *[14] and *Artemia *sp. (Anostraca) were exclusive of deeper layers in the lakes studied. The cyst assemblage composition demonstrated highly significant differences among lakes (ANOSIM, R value = 0,947, P***). This result is clearly shown by MDS plot (Figure 3): samples (represented by cyst types and abundances) from each hypersaline lake grouped separately one another.

The SIMPER procedure associated with MDS of all samples identified the main species responsible for the biotic characterisation of each lake [see Additional file 2]. *Artemia *sp. eggs characterised the "cyst bank" of Nartë saltworks (SIMPER, similarity percentage: 73%), *A. urmiana *eggs characterised the Koyashskoe lake (SIMPER, similarity percentage: 69%), Copepoda Calanoida eggs (unidentified) and *Hexarthra fennica *eggs characterised the Khersonesskoe lake (SIMPER, similarity percentage: 84%), while Turbellaria cocoons and *Hexarthra fennica *eggs were the main responsible for the average similarity of Vecchia Salina, Pantano Grande and Pantano Roveto in Italy (SIMPER, similarity percentage: 72%, 70% and 75%, respectively).

**Figure 4:**
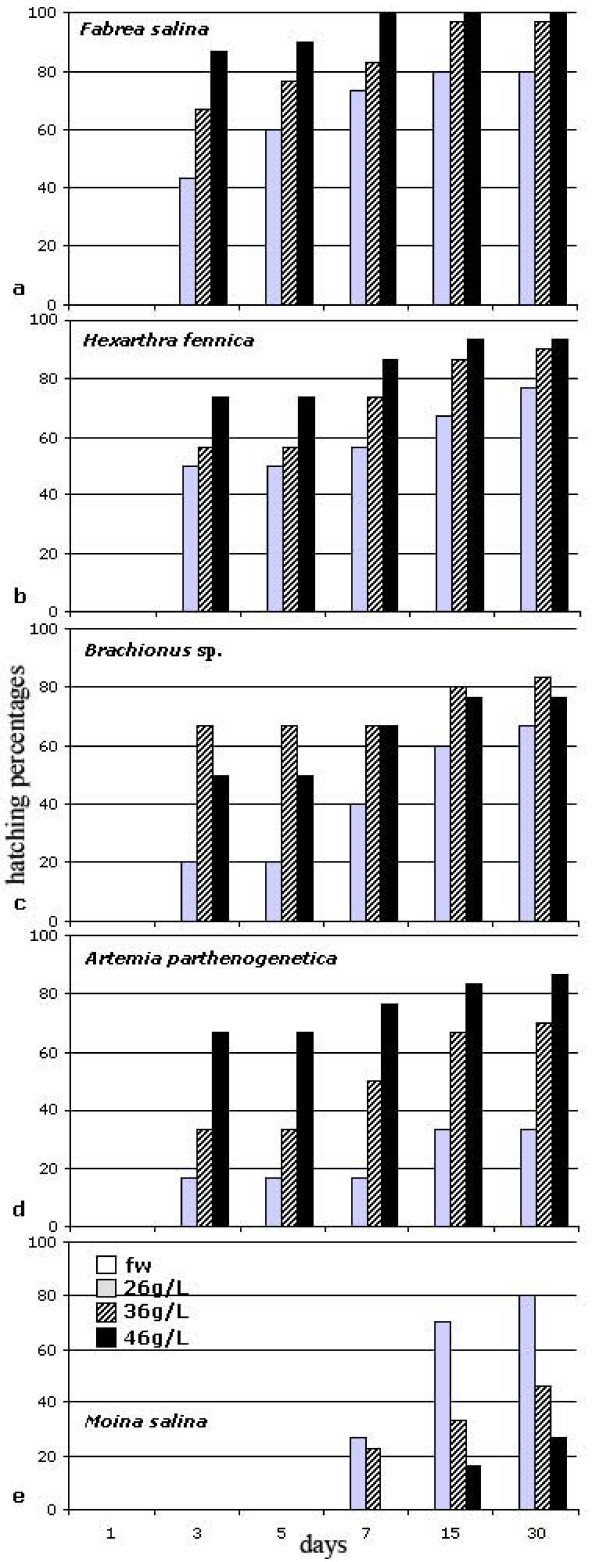
**Best hatching patterns of some resting stages submitted to laboratory controlled conditions: a) *Fabrea salina *cysts; b) *Hexarthra fennica *eggs; c) *Brachionus *sp. eggs; d) *Artemia parthenogenetica *eggs; e) *Moina salina *ephippia**. The values reported on the *x*-axis correspond to the storage time (in days); the *y*-axis represents the hatching percentages.

Laboratory experiments under controlled conditions let us to estimate the hatching success of some species (Figure 4). In *Fabrea salina *(Ciliophora) 100% of hatching was obtained in equinox conditions (12 h light, 12 h dark; at a salinity of 46‰ after 1 week). In *Hexarthra fennica *(Rotifera) the maximum hatching (93%) was obtained in summer light conditions (14 h light; 10 h dark; at a salinity of 46‰ after 2 weeks). In *Brachionus *sp. (Rotifera) a hatching peak (66,7%) occurred after 3 days at a salinity of 46‰, under summer light conditions. *Artemia *sp. (Crustacea Anostraca) showed the maximum hatching-rate under summer conditions (67% at a salinity of 36‰ after 3 days). *Moina salina *(Crustacea Cladocera) showed an hatching peak (70%) occurring at a salinity of 26‰ after 2 weeks, in summer light conditions.

## Discussion and conclusion

The study of cyst bank of 6 hypersaline lakes allowed us to give a dimension to the unexpressed, potential biodiversity inhabiting such a typology of stressed environments. A sensible portion of the biodiversity of each lake rests during most of the year, and only a portion of the species present result active in the water column at each date. The accumulation of cysts of different species, generations and genotypes with variable hatching rates leads to a mixed cyst bank more bio-diverse than the active community sampled at any one moment.

Available information coming from the Vecchia Salina [10] reported a total of 16 species in the water column (deriving from a sampling effort which lasted over two years). The number of cyst types (17) here reported from the bottom sediments of the same environment is lower than that (24) reported by the same authors, but in that case the investigation was carried out on a total of 14 cores collected in 14 different sites of the lake, while in the present study only the central area of the lake was concerned by the collection of just 3 replicate cores.

It is evident that such a light sampling effort (3 sediment cores at one sampling date) devoted to the sediments gives more information than a two-year period of sample collection in the water. Nevertheless, we cannot ignore the fact that some species do not supply the sediment cyst bank but overcome adverse periods differently (e.g. migrating as insects do, or resting as sub-adults, as some harpacticoids do). Hence the analysis of cyst banks cannot still completely substitute the traditional investigation on active stages. It could be proposed, however, as an indispensable additional source of information to correctly evaluate the biodiversity of water environments [10,13]. A greater diversity of zooplankters are represented in the egg banks in comparison to that present in the current water column, these are dormant phases of the zooplankters life cycle and favourable hatching conditions for those unique cohorts may or may not occur within a given lake. The egg banks stored in sediments of each lake represent its real potential diversity. This does not diminish their importance over time because, as environmental conditions in a lake change its appropriate zooplankter assemblage, it will be more (or less) dominant.

As regards the species composition of each assemblage, it can be noted that Koyashskoe and Nartë were the poorest, while Vendicari pools and Vecchia Salina saltworks were the richest lakes.

Some information about salinity [see Additional file 3] suggests that this could be due to the high salt concentrations characteristic of the first waters. The biodiversity of saline habitats, in fact, seems to be inversely correlated with the salinity value [1,15]. As regards the species composition, we can note that both the environments of Vendicari (Pantano Grande and Pantano Roveto) do not host *Artemia *cysts, while they are the only habitats where diaptomid eggs have been found. As in the case described by [16], despite the proximity to the sea, the salt is probably not of marine but of athalassohaline origin (underground brines). It has been proved that the quality of salt content affects the biological community [6,7], and a chemical analysis of dissolved anions in the Vendicari lakes water will be the necessary future information to be collected to confirm this rule. The sediments of both Vendicari lakes (Pantano Grande and Pantano Roveto) sampled during the dry season (September 2005), did not show the presence of *Artemia *cysts, while there have been recognized eggs of other Anostraca (*Phallocryptus *sp.) and Calanoida (*Arctodiaptomus *sp.). Although the two lakes are close to the sea, this taxonomic composition of egg bank suggests their salinity is not entirely of marine origin, but influenced by continental inputs.

The wide hatching patterns observed for most of the tested species is thought to be an obligatory adaptation to the extreme variability of the habitat. Hatching tests affirmed that the 5 tested species inhabiting the present lakes dislike freshwater conditions. Hatching has been found to be highly variable, even among cysts coming from the same sediment level, as well as among different layers of each core, probably due to the need to spread the risk of non successful hatching over many attempts, according to the bet hedge theory of [17,18] to ensure the persistence of populations in unpredictably stressed environments. Indeed, most of cyst morphotypes here considered belong to species that live in environments with a high level of stochasticity [19], a condition in which bet-hedging is expected to evolve [20].

Cyst banks form an essential component of plankton ecology. As cyst banks integrate seasonal and year-to-year variations in environmental conditions, they represent the total species and genetic diversity in any community better than the active component sampled at any one time. Cyst banks can be considered the archive of the local habitat, and overlooking the composition of the cyst bank in the study of biodiversity and biogeography may result in erroneous patterns and interpretations of the underlying processes.

## Methods

### Study site

**Figure 5:**
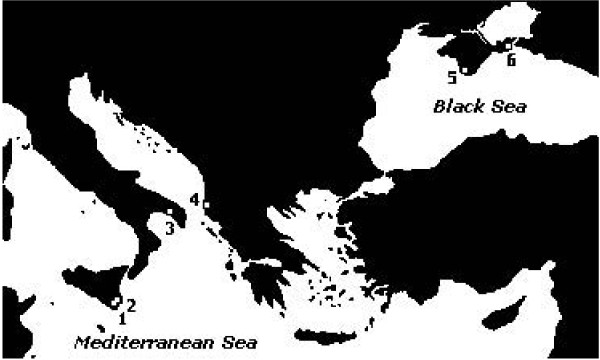
**Geographical location of the sampling sites in the South-Eastern European Region**. 1,2: *Pantano Grande *and *Pantano Roveto *at Vendicari Nature Reserve (Italy); 3: *Vecchia Salina *at Torre Colimena (Gulf of Taranto, Apulia, Italy); 4: *Nartë *saltworks (Albania); 5: *Khersonesskoe *lake (Crimea, Ucraine); 6: *Koyashskoe *lake (Crimea, Ucraine).

The research interested, from April 2004 to September 2006, Khersonesskoe (44°35'12"N; 33°24'00"E) and Koyashskoe (45°02'31"N; 36°12'20"E) lakes in the Crimea peninsula (Ukraine), Nartë (40°32'13"N; 19°25'48"E) saltworks (Southern Albania), Vecchia Salina (40°18'06"N; 17°43'56"E) at Torre Colimena (Gulf of Taranto, Apulia, Italy), Pantano Grande and Pantano Roveto (36°48'29"N; 15°06'02"E) at Vendicari Nature Reserve (Sicily, Italy) (Figure 5).

Collections of resting stages from the sediments of the 2 contiguous lakes at Vendicari (Sicily, Italy) allowed to compare results coming from nearby habitats with those coming from very distant ones (e.g. Italian and Crimean lakes).

### Sampling procedures

Samplings were collected during summer months corresponding to the minimum water level, or even its absence to take only samples not submerged. Three replicate sediment cores (diameter, 7.5 cm; depth, 6 cm) were obtained from each lake by using a core sampler (20 cm length) and stored at 4°C in a refrigerator for 1 year. For the analysis, each sediment core was cut into 3 cm thick layers. Each layer was ultrasonified to break the larger particles of sediment and then sieved at two mesh sizes (212 and 45 μm). The sediment collected by both sieves was centrifuged at 1,090 g in a 1:1 sucrose-distilled water solution for 3 min. The supernatant derived from the centrifugation of the two sieve fractions was analysed to separate cysts.

Cysts were reported as number per 100 cc of sediment. The most abundant resting stages (2 types for the fraction >212 μm; 3 types for the fraction >45 μm) were used in hatch experiments in the laboratory. Sets of 30 cysts of each morphotypes, taken from each layer (from the superficial to the deepest one), were stored in 3 cc wells raised with 2 cc of original water filtered at 0,45 μm. To avoid bacterial growth, in each well, 20 μl of an antibiotic mix (streptomycin/penicillin 1:1) was added. Resting stages were submitted to different storage conditions in thermostatic rooms (an "equinox" simulation, with 13°C and 12 hL:12 hD photoperiod and a "early summer" simulation, with 24°C and 14 hL:10 hD photoperiod) at 4 different salinity values (46‰, 36‰, 26‰, freshwater) obtained by diluting the original-site water. Hatching plates were checked daily to test the presence of active stages, which were counted and removed for identification.

The related water column, on each lake, has been sampled in different periods of the year (at least 2 seasons), and in different years (between 2002 and 2006) to compare its faunal composition to each other. Zooplankton samples were collected monthly (three replicates), with two plankton nets (mouth diameter, 25 cm; length, 65 cm; mesh size, 200 μm and 50 μm) towed horizontally, equipped with a water-flow meter at the mouth. The Italian hypersaline lake Vecchia Salina and Crimean ones were already studied in the past (see data in [10] and [16], respectively).

### Data analysis

Data were analysed by multivariate statistical techniques with a non-parametric approach because of the wide disparity in density of some cysts in different lakes. The significance of the spatial variation in "cyst banks" composition was tested using a One-Way Analysis of Similarities for replicated data (ANOSIM) routine in PRIMER (Plymouth Routines In Multivariate Ecological Research) version 6β R6 (PRIMER-E) [21].

For multivariate analyses, the absolute densities of each morphotypes were fourth root transformed, to severely down-weight the importance of the very abundant species so allowing the less dominant, and even the rare morphs, to play some role in determining similarity among samples.

Stress values were shown for each MDS plot to indicate the goodness of representation of differences among samples [22]. A One-Way similarity percentages procedure (PRIMER SIMPER routine, Clarke [22]) was used in order to obtain the percentage contribution that each *taxon *provided to Bray-Curtis similarities measures. A cut-off criterion was applied to allow the identification of a subset of species whose cumulative percentage contribution reached 80% of similarity value. SIMPER analysis consented to identify the species responsible for the biological characterisation of the "seed banks" stored in each investigated lake.

## Abbreviations

VEN: Vendicari ponds; PGR: Pantano Grande; PRV: Pantano Roveto; TCL: Torre Colimena (Vecchia Salina) saltworks; NRT: Nartë saltworks; KHE: Khersonesskoe lake; KOY: Koyashskoe lake; hL: hours of light; hD: hours of dark; sim%: similarity percentage.

## Competing interests

The authors declare that they have no competing interests.

## Authors' contributions

SM participated in the design of the study, executed cyst collection in all the lakes, performed the statistical analysis and is responsible for the first draft of the manuscript. GB conceived of the study, participated in its design and coordinated all the steps, comprising the discussion of results.

## Supplementary Material

Additional file 1 **Table S1. **Number of taxonomic groups representing the total biodiversity (realised and potential) of the different lakes investigated. Click here for file

Additional file 2 **Table S2.** Cyst categories contributing most (80% cut off) to the biotic characterisation of each lake (av.ab. = average abundance; av.sim. = average similarity; contrib.% = contribution percentage; cum.% = cumulative percentage). Click here for file

Additional file 3 **Table S3.** Hypersaline lakes considered in the present study (listed in longitudinal order): salinity (‰) and depth (m) values. Click here for file
