# Supplementary material for: Egg banks in hypersaline lakes of the South-East Europe
Source: Saline Syst. 2009 Mar 17;5:3. doi: 10.1186/1746-1448-5-3 (PMC2662865; doi:10.1186/1746-1448-5-3)
Supplement: Additional file 1 — Table S1. Number of taxonomic groups representing the total biodiversity (realised and potential) of the different lakes investigated. [file 1746-1448-5-3-S1.doc]

Table S1

| **Lake** | **Active stages** | **Dormant stages** |
| --- | --- | --- |
| Pantano Grande, Vendicari (Italy) | 6 | 15 |
| Pantano Roveto, Vendicari (Italy) | 7 | 15 |
| Torre Colimena (Italy) | 16 | 17 |
| Nartë (Albania) | 2 | 8 |
| Khersones (Ukraine) | 5 | 14 |
| Koyash (Ukraine) | 3 | 5 |
