# Supplementary material for: Egg banks in hypersaline lakes of the South-East Europe
Source: Saline Syst. 2009 Mar 17;5:3. doi: 10.1186/1746-1448-5-3 (PMC2662865; doi:10.1186/1746-1448-5-3)
Supplement: Additional file 2 — Table S2. Cyst categories contributing most (80% cut off) to the biotic characterisation of each lake (av.ab. = average abundance; av.sim. = average similarity; contrib.% = contribution percentage; cum.% = cumulative percentage). [file 1746-1448-5-3-S2.doc]

Table S2

Pantano Grande - Vendicari (Sicily, Italy) Av.Sim=69,96%

| taxa | av.ab. | av.sim. | contrib.% | cum.% |
| --- | --- | --- | --- | --- |
| Turbellaria eggs | 5,54 | 10,26 | 14,66 | 14,66 |
| Bivalvia undetermined 1 | 5,61 | 8,23 | 11,76 | 26,43 |
| Ostracoda undetermined | 5,73 | 8,05 | 11,50 | 37,93 |
| *Hexarthra fennica* cysts | 5,17 | 6,86 | 9,81 | 47,74 |
| Gastropoda undetermined 2 | 3,55 | 5,50 | 7,86 | 55,61 |
| *Arctodiaptomus salinus* eggs | 4,53 | 4,63 | 6,62 | 62,23 |
| Brachionidae eggs | 3,64 | 4,15 | 5,93 | 68,16 |

Pantano Roveto - Vendicari (Sicily, Italy) Av.Sim=75,74%

| taxa | av.ab. | av.sim. | contrib.% | cum.% |
| --- | --- | --- | --- | --- |
| Turbellaria eggs | 4,76 | 11,07 | 14,62 | 14,62 |
| *Hexarthra fennica* eggs | 5,47 | 10,19 | 13,45 | 28,07 |
| Ostracoda undetermined | 3,23 | 6,94 | 9,17 | 37,24 |
| *Fabrea salina* cysts | 4,31 | 6,47 | 8,54 | 45,78 |
| Bivalvia undetermined 1 | 2,71 | 5,52 | 7,29 | 53,07 |
| Foraminiferida undetermined 2 | 2,87 | 5,43 | 7,17 | 60,24 |
| *Ptygura* sp. eggs | 2,94 | 5,02 | 6,62 | 66,86 |

Torre Colimena (Apulia, Italy) Av.Sim=72,19%

| taxa | av.ab. | av.sim. | contrib.% | cum.% |
| --- | --- | --- | --- | --- |
| Turbellaria eggs | 5,98 | 12,97 | 17,97 | 17,97 |
| *Hexarthra fennica* eggs | 4,28 | 8,32 | 11,53 | 29,50 |
| Cyst type 1 | 3,61 | 8,15 | 11,30 | 40,80 |
| *Artemia parthenogenetica* eggs | 3,11 | 6,87 | 9,52 | 50,31 |
| *Phallocryptus spinosa* eggs | 3,08 | 6,59 | 9,13 | 59,44 |
| *Ptygura* sp. eggs | 2,74 | 5,54 | 7,67 | 67,11 |

Nartë (Albania) Av.Sim=73,04%

| taxa | av.ab. | av.sim. | contrib.% | cum.% |
| --- | --- | --- | --- | --- |
| Foraminiferida undetermined 4 | 4,20 | 22,02 | 30,14 | 30,14 |
| Cyst type 3 | 3,07 | 18,19 | 24,90 | 55,05 |
| *Artemia parthenogenetica* eggs | 2,08 | 12,31 | 16,86 | 71,90 |

Khersonesskoe (Crimea, Ukraine) Av.Sim=83,75%

| taxa | av.ab. | av.sim. | contrib.% | cum.% |
| --- | --- | --- | --- | --- |
| Copepoda undetermined eggs | 7,01 | 16,28 | 19,44 | 19,44 |
| *Hexarthra fennica* eggs | 5,29 | 12,31 | 14,70 | 34,14 |
| Ostracoda undetermined | 5,65 | 11,43 | 13,65 | 47,79 |
| Turbellaria eggs | 4,32 | 9,24 | 11,03 | 58,82 |
| Brachionidae eggs | 3,57 | 7,88 | 9,41 | 68,23 |
| *Artemia parthenogenetica* eggs | 3,35 | 7,12 | 8,50 | 76,73 |

Koyashskoe (Crimea, Ukraine) Av.Sim=69,04%

| taxa | av.ab. | av.sim. | contrib.% | cum.% |
| --- | --- | --- | --- | --- |
| *Artemia urmiana* eggs | 4,75 | 28,68 | 41,54 | 41,54 |
| Cyst type 2 | 2,12 | 13,77 | 19,94 | 61,49 |
| Turbellaria eggs | 1,95 | 13,00 | 18,83 | 80,32 |
