# Supplementary material for: Egg banks in hypersaline lakes of the South-East Europe
Source: Saline Syst. 2009 Mar 17;5:3. doi: 10.1186/1746-1448-5-3 (PMC2662865; doi:10.1186/1746-1448-5-3)
Supplement: Additional file 3 — Table S3. Hypersaline lakes considered in the present study (listed in longitudinal order): salinity (‰) and depth (m) values. [file 1746-1448-5-3-S3.doc]

Table S3.

| **Lake** | **Salinity min-max (‰)** | **Depth max (m)** |
| --- | --- | --- |
| Pantano Grande, Vendicari (Italy) | 18-280 | 0.7 |
| Pantano Roveto, Vendicari (Italy) | 35-290 | 0.6 |
| Torre Colimena (Italy) | 26-296 | 0.8 |
| Nartë (Albania) | 180-270 | 0.6 |
| Khersones (Ukraine) | 35-120 | 1.0 |
| Koyash (Ukraine) | 235-365 | 1.2 |
